# Accelerating the impact of artificial intelligence in mental healthcare through implementation science

**DOI:** 10.1177/26334895221112033

**Published:** 2022-07-11

**Authors:** Per Nilsen, Petra Svedberg, Jens Nygren, Micael Frideros, Jan Johansson, Stephen Schueller

**Affiliations:** 1 4566 Linköping University, Linkoping, Sweden; 2 166447 Halmstad University School of Health and Welfare, Halmstad University, Halmstad, Sweden; 3 3695 Region Halland, Halmstad, Sweden; 4 Psychological Science, 8788 University of California Irvine, Irvine, CA, USA

**Keywords:** mental health services, implementation, artificial intelligence

## Abstract

**Background:**

The implementation of artificial intelligence (AI) in mental healthcare offers a potential solution to some of the problems associated with the availability, attractiveness, and accessibility of mental healthcare services. However, there are many knowledge gaps regarding how to implement and best use AI to add value to mental healthcare services, providers, and consumers. The aim of this paper is to identify challenges and opportunities for AI use in mental healthcare and to describe key insights from implementation science of potential relevance to understand and facilitate AI implementation in mental healthcare.

**Methods:**

The paper is based on a selective review of articles concerning AI in mental healthcare and implementation science.

**Results:**

Research in implementation science has established the importance of considering and planning for implementation from the start, the progression of implementation through different stages, and the appreciation of determinants at multiple levels. Determinant frameworks and implementation theories have been developed to understand and explain how different determinants impact on implementation. AI research should explore the relevance of these determinants for AI implementation. Implementation strategies to support AI implementation must address determinants specific to AI implementation in mental health. There might also be a need to develop new theoretical approaches or augment and recontextualize existing ones. Implementation outcomes may have to be adapted to be relevant in an AI implementation context.

**Conclusion:**

Knowledge derived from implementation science could provide an important starting point for research on implementation of AI in mental healthcare. This field has generated many insights and provides a broad range of theories, frameworks, and concepts that are likely relevant for this research. However, when taking advantage of the existing knowledge basis, it is important to also be explorative and study AI implementation in health and mental healthcare as a new phenomenon in its own right since implementing AI may differ in various ways from implementing evidence-based practices in terms of what implementation determinants, strategies, and outcomes are most relevant.

**Plain Language Summary:** The implementation of artificial intelligence (AI) in mental healthcare offers a potential solution to some of the problems associated with the availability, attractiveness, and accessibility of mental healthcare services. However, there are many knowledge gaps concerning how to implement and best use AI to add value to mental healthcare services, providers, and consumers. This paper is based on a selective review of articles concerning AI in mental healthcare and implementation science, with the aim to identify challenges and opportunities for the use of AI in mental healthcare and describe key insights from implementation science of potential relevance to understand and facilitate AI implementation in mental healthcare. AI offers opportunities for identifying the patients most in need of care or the interventions that might be most appropriate for a given population or individual. AI also offers opportunities for supporting a more reliable diagnosis of psychiatric disorders and ongoing monitoring and tailoring during the course of treatment. However, AI implementation challenges exist at organizational/policy, individual, and technical levels, making it relevant to draw on implementation science knowledge for understanding and facilitating implementation of AI in mental healthcare. Knowledge derived from implementation science could provide an important starting point for research on AI implementation in mental healthcare. This field has generated many insights and provides a broad range of theories, frameworks, and concepts that are likely relevant for this research.

Mental health disorders are prevalent in society and are a serious public health problem. In 2001, the World Health Organization estimated that 450 million people worldwide had such problems (World Health Organization, 2001). Approximately 75% of mental disorders emerge before the age of 25 years (Jones, 2013), and it is expected that 29% of people will experience a mental disorder at least once in their lifetime (Barrett et al., 2017). Mental illness restricts individuals’ abilities to function, engage in daily activities, and maintain social relationships, causing significant suffering to individuals and their families (Fricchione et al., 2012; Rosenfeld et al., 2021).

Despite the prevalence and severity of mental illness, there are many barriers to achieving optimal detection, prevention, treatment, and monitoring of mental health disorders (Schueller & Torous, 2020). Availability of mental healthcare remains limited. Many people with mental illnesses receive no mental healthcare, and even those who do rarely receive evidence-based mental healthcare (Alegría et al., 2018; Kilbourne et al., 2018). There is a shortage of psychiatrists, psychologists, nurses, and social workers (Fricchione et al., 2012; Liu et al., 2017); almost half of the world's population live in countries where there is less than one psychiatrist per 100,000 people (World Health Organization, 2017). This shortage of mental healthcare professionals restricts care opportunities, resulting in long waiting lists, delays, and multiple, diverse contacts before appropriate care is obtained (MacDonald et al., 2018; Westberg et al., 2020).

Even when providers exist, studies have documented accessibility barriers, including concerns about the cost, transportation or inconvenience, problems recognizing mental illness symptoms (i.e., poor mental health literacy), and lack of awareness of care options (Gulliver et al., 2010; Pedersen & Paves, 2014). Attitudinal barriers also prevent people from seeking mental healthcare, including the belief that it will not help and a preference for self-reliance. Public stigma against mental health disorders and those who receive care is a common concern that also functions as a barrier (Gulliver et al., 2010; Wainberg et al., 2017). As such, even where care is hypothetically available, few in need of such care actually receive it.

The implementation of artificial intelligence (AI) in mental healthcare offers a potential solution to some of the problems with the availability, attractiveness, and accessibility of mental healthcare services (Bickman, 2020; European Institute of Innovation and Technology, 2020; Lee, 2019; Topol, 2019). AI generally refers to a computerized system (hardware or software) that is able to perform tasks or reasoning processes that we usually associate with intelligence in a human being (European Union, 2018). AI applied to mental healthcare includes techniques to improve the ability to detect and predict various conditions, with implications for screening, assessment, and clinical decision-making (Graham et al., 2019). AI can also be used to improve treatments by identifying treatments or treatment elements most likely to provide benefit, thus promoting personalized mental healthcare (Bickman, 2020), or can be incorporated into digital interventions, such as smartphone apps, to create novel interventions, thus reducing reliance on professionals and increasing scalability (D’Alfonso, 2020).

While AI applications have become increasingly commonplace in many areas of business and society, the potential of using AI in mental healthcare has not been realized to date. Still, there are examples of AI applications that have been implemented and demonstrated the promise of AI in mental health practice, such as leveraging existing data streams or creating new interventions (Lee et al., 2021) and platforms to enable therapists to receive data-led insights about each patient during the assessment stage of treatment (Ieso Health, 2022). An example is the implementation of a suicide prevention AI application, Recovery Engagement, and Coordination for Health-Veterans Enhanced Treatment (REACH VET) intended to identify individuals in the Veterans Health Administration electronic health records most likely to die from suicide. Using an interactive dashboard, program coordinators receive information and communicate with clinicians who re-evaluate treatment strategies and conduct outreach to initiate care enhancements (McCarthy et al., 2021). There are also numerous mental health self-assessment AI applications available, e.g., the Ellie digital avatar that was initially developed to help war veterans struggling with depression and Post Traumatic Stress Syndrome, the BioBase app that leverages AI to interpret sensor data from a wearable as well as apps like Woebot and Elomia that target individuals with mild symptoms of anxiety or depression (Lavrentyeva, 2021; Raibagi, 2020). AI mental health applications such as these have produced considerable enthusiasm for development and implementation, including a substantial investment in mental health startups using AI (Shah & Berry, 2021) and pilots in healthcare systems to consider opportunities (Universal Health Services, 2020). Despite examples of AI use in mental healthcare practice, Davenport and Kalakota (2019) argue that the greatest challenge to AI “is not whether the technologies will be capable enough to be useful, but rather ensuring their adoption in daily clinical practice.” Seneviratne et al. (2019) suggest that AI implementation in healthcare is “the elephant in the room.”

Despite the promise of AI applications in mental healthcare, considerable knowledge gaps still exist, including how to best implement and use these innovations to add value to healthcare services, providers, and consumers. Implementation science knowledge may be useful for understanding and addressing the challenges of implementing AI in mental healthcare. The aim of this paper is to review the extant literature to identify challenges and opportunities for the use of AI in mental healthcare and describe key insights from implementation science of potential relevance to understand and facilitate AI implementation in mental healthcare. To this end, we conducted a selective literature review of articles concerning AI in mental healthcare and implementation science.

## AI in Mental Healthcare

Mental healthcare differs in many ways from medicine, which contributes to the need to consider unique challenges and opportunities related to its implementation. Unlike the diagnosis of a physical condition that can be based on laboratory tests or physiologic measurements, diagnoses of mental illness typically rely on patients’ self-reported information and mental health professionals’ judgment (Rosenfeld et al., 2021; Su et al., 2020). Mental health professionals emphasize the importance of the relational and observational aspects in therapeutic practice, such as forming a therapeutic alliance with the patient and directly observing patient behaviors and emotions (Graham et al., 2019). A multi-country survey by Doraiswamy et al. (2020) showed that mental health professionals believed that AI can be used for documenting and synthesizing information, but they did not think it can replace the interaction between patient and professional. However, there is considerable optimism among AI and mental health researchers that mental healthcare can benefit greatly from the use of AI (Barrett et al., 2017; D’Alfonso, 2020; Graham et al., 2019; Mohr et al., 2017; Rosenfeld et al., 2021; Su et al., 2020; Topol, 2019).

AI has a potentially important role in improving our understanding of mental illness to achieve improved detection, prevention, treatment, and monitoring of mental health disorders (Graham et al., 2019; Lee et al., 2021; Su et al., 2020). In these areas, the application of AI in mental health can broadly be classified into opportunities for selection and for assessment.

## Opportunities for Selection

Opportunities for selection involve identifying the patients most in need of care or the interventions that might be most appropriate for a given population or individual. In the service of selection, AI applications based on data from large populations can help identify previously unidentified correlations. Such models can help detect unobservable mental health states, analyze complex problems, and monitor trends and thresholds of key mental health indicators. As examples, AI applied to social media data can detect individuals who are at higher risk for suicide (Coppersmith et al., 2018). AI has also been used to determine who might be most likely to benefit from cognitive behavioral or psychodynamic therapy in routine healthcare settings (Schwartz et al., 2021).

## Opportunities for Assessment

Opportunities for assessment include supporting a more reliable diagnosis of psychiatric disorders and ongoing monitoring and tailoring during the course of treatment to maximize opportunities for response. In the service of assessment, AI offers opportunities to combine bio-psycho-social data to create novel assessment streams such as the determination of mental health states through passive smartphone data (Mohr et al., 2017). Applied to evaluation of interventions, AI can support understanding of what treatment elements most have an impact on improvement (Ewbank et al., 2020) and might assist in monitoring elements during therapy, such as emotion (Tanana et al., 2021) or symptom change that might facilitate better measurement-based care (Huckvale et al., 2019) or fidelity to evidence-based practices in ways that can help clinicians improve or adapt their techniques (Flemotomos et al., 2021). In the service of these multiple areas, AI offers the opportunity to use novel or larger data streams which can facilitate a more reliable diagnosis of psychiatric disorders and support patients and providers in making more informed shared decisions as part of a more person-centered care (Aafjes-van Doorn et al., 2021; Graham et al., 2019; Su et al., 2020).

## Implementation of AI in Mental Healthcare

Much AI research and development (R&D) has followed a generic R&D pipeline, from ideas and hypotheses to AI applications used in routine practice, with progress varying between areas of AI use. The pipeline begins with hypotheses and in silico experiments (i.e., performed on a computer or via computer simulation), progressing to proof-of-concept (prototype) projects to document the feasibility of the AI application, before studies are carried out to evaluate the efficacy and effectiveness of the application when it is deployed in practice. The final step of this pipeline is implementation of the application as standard practice in routine health and mental healthcare (Stead, 2018).

It is firmly established in implementation and innovation research that novel technologies are often resisted by healthcare professionals, contributing to a slow and variable uptake (Safi et al., 2018; Whitelaw et al., 2020). Several types of implementation challenges (i.e., barriers and facilitators) at different levels have been documented in AI research. Here, we provide an overview of challenges at different levels: organizational and policy, providers, patients, and technical. The challenges are likely interdependent, making it difficult to assess the relative importance of various challenges or identify which challenges might be most difficult to overcome. However, dividing the challenges into these levels provides an opportunity for implementation frameworks to support understanding and addressing them given the considerations of such levels in many such frameworks.

## Organizational and Policy Challenges

There are many organization- and policy-level challenges to AI implementation in healthcare. Regulatory concerns include governance of autonomous AI systems, responsibility and accountability issues, paucity of industry standards for use of AI use and assessment of safety, and inadequate or non-existing clinical and economic impact measurement (Buch et al., 2018; Esmaeilzadeh, 2020; Horgan et al., 2019; Liyanage et al., 2019). The literature does not expound on appropriate strategies to address various barriers, but He et al. (2019) recommend the use of specific task force committees in healthcare organizations to work specifically with AI issues.

A major hurdle with regard to the organization and policy levels is the lack of clear and consistent guidelines regarding the application of AI in healthcare generally, and mental healthcare consistently. In the United States, regulatory issues with regard to AI in healthcare are still being defined by the U.S. Food and Drug Administration. The European Union has developed Ethical Guidelines for Trustworthy AI and is now working on turning these into law. In the proposed legislation, most healthcare applications will likely be considered high-risk applications, with significant regulation associated (European Union, 2019). In other countries, standards for the use of digital mental health products (Brown et al., 2021) have not addressed the additional complications that AI applications bring. Therefore, given the current lack of regulation, new shifts in regulatory guidelines could have considerable impact on implementation and organization- and policy-level challenges.

## Challenges for Providers

At the individual level, professionals must have access to education and training to learn new skills as AI users. They need tools to be able to train AI systems to set them up to perform specified tasks, and they must be able to understand and interpret AI outputs (Buch et al., 2018; He et al., 2019; Mistry, 2019). Integrating AI into clinical practice will require professionals to adapt their practice and actively engage with AI to achieve “a partnership where the machine predicts, and the human explains and decides on action” (Verghese et al., 2018). However, research has documented considerable skepticism among healthcare professionals on using AI (Lee, 2019; Liyanage et al., 2019). Reflecting on the AI implementation challenges, Kelly et al. (2019) concluded that “human barriers to adoption [of AI] are substantial.”

## Challenges for Patients

There may also be mistrust among patients in mental healthcare, with concerns about technical aspects (e.g., communication barriers), ethical issues (e.g., privacy), and regulatory aspects (e.g., unregulated standards) (Esmaeilzadeh, 2020). These concerns relate to AI in general, but they might be even more pronounced in mental healthcare. For example, psychiatric diagnoses are defined by criteria related to daily life and are formulated in ordinary language that is often easy for patients to comprehend. However, it may be more difficult to understand and accept that signs indicating, for example, depression, suicidal ideation, or social anxiety, are identified by AI through analysis of data contained in a patient's medical record (Esmaeilzadeh, 2020), smartphone (Gong et al., 2019) or on social media (Lekkas et al., 2021), potentially leading to communication barriers between patient and mental healthcare professionals. Patients might also experience various privacy concerns because the use of AI often requires substantial datasets to provide insights (Uusitalo et al., 2021). Patients might be more willing to provide mental health data based on the different proposed uses or recipients of that data (Nicholas et al., 2019), but in many applications of AI, the ultimate use of that data may not be known when it is first collected.

## Technical Challenges

Technical challenges to AI implementation include the lack of transparency of data and algorithms, safety of AI recommendations, and complexities in interpreting outputs and dealing with responsibilities for the outcomes of decisions based on AI-generated information. Concerns have been raised about data being too fragmented and siloed in many systems that can be difficult to bring together. Data may also be unusable due to a lack of data that meets precise requirements or is sufficiently labeled. Thus, there may paradoxically be both too much data (i.e., datasets are large but lack meaningful information or sufficient labels) and too little data (i.e., data that are not appropriate or sufficient for specified purposes). Just because a dataset is large does not mean it will be applicable to a given problem, especially as too much similar data might not provide much additional value. Furthermore, when Electronic Health Record data are used, mental health data tend to be less structured and therefore more messy (Dawoodbhoy et al., 2021). When datasets are small, they may not be representative and fail to generalize when applied more broadly. This might introduce algorithm bias and models that fail to predict in specific populations (Brodwin & Ross, 2021; Coley et al., 2021).

Furthermore, software must be continuously updated and evaluated with regard to applicability for datasets that might vary from the data on which models have been trained and validated. Also, sufficient hardware to support any provider interface with models is necessary (Buch et al., 2018; Horgan et al., 2019; Keane & Topol, 2018; Kelly et al., 2019). Given that mental healthcare settings are under resourced, sufficient technical infrastructure may not be present.

Methods used to capture the data need to be sufficiently standardized and robust to prevent negative effects from handling or data management (Geirhos et al., 2020). Another technical challenge is that the data used to train an AI application must be relevant for the actual application. For example, if an AI system is used to interpret facial expressions, it needs to be trained on examples representative for the actual target group because social communication might differ depending on factors such as age, culture, or region (Kim et al., 2019). Some of the technical challenges may be particularly relevant for mental healthcare. AI algorithms based on neural networks require many different examples, hundreds or thousands, to learn a particular diagnosis or treatment (Cho et al., 2016). This means that an AI system based on neural networks will often struggle when confronted with a rare, albeit distinct and well described, diagnosis. For a well-trained human, on the other hand, one rich example described in research might suffice.

## Using Implementation Science to Understand Implementation of AI

In recognition of the challenges in implementing AI, it may be important to draw on implementation science knowledge to understand and facilitate implementation of AI in mental healthcare. Research in implementation science has generated many insights concerning implementation in various healthcare settings, establishing the importance of considering and planning for implementation from the start, the progression of implementation through different stages, and the appreciation of influences from multiple levels of the healthcare system and beyond (Nilsen, 2015). So-called hybrid designs are used in implementation science to accelerate the research-to-practice process. This design allows researchers to investigate the effects of both a clinical (patient-level) intervention and the implementation of this intervention (Curran et al., 2012). This study design may be relevant to investigate whether or the extent to which various AI applications warrant implementation in mental health.

Numerous determinant frameworks and implementation theories have been developed in implementation science to understand and explain how different barriers and facilitators impact on implementation (Nilsen, 2015), e.g., Consolidated Framework of Implementation Research (Damschroder et al., 2009), Promoting Action on Research in Health Services (PARIHS) and integrated-PARIHS (Harvey & Kitson, 2016), Exploration Preparation Implementation Sustainability (Aarons et al., 2011), Theoretical Domains Framework (Michie et al., 2014), Capability Opportunity Motivation Behaviour (COM-B) (Michie et al., 2011), and Normalization Process Theory (May & Finch, 2009), all of which are widely used in the field. Knowledge about determinants of implementation outcomes provides input for selecting the most effective strategies to overcome barriers and/or harness facilitators. Together these frameworks and theories describe five interdependent domains of influence on implementation: (1) effectiveness of the strategy used to support implementation; (2) attributes of the implemented practice (i.e. an intervention, programme, or service), e.g., its perceived complexity and compatibility with previous practices; (3) features of the adopters, e.g., the providers’ attitudes, beliefs, and motivation concerning the implemented practice; (4) features of the patients or recipients of the implemented practice, e.g., their preferences; and (5) contextual influences, e.g., the culture, leadership, and other collective-level influences on the adopters (Nilsen, 2015).

AI implementation research should explore the extent to which the barriers and facilitators to AI implementation overlap with those identified in studies of implementation of evidence-based practices. Furthermore, because AI is not one single technology but rather many different ones (Davenport & Kalakota, 2019; Shaw et al., 2019), it is also important to investigate different AI applications with regard to these barriers and facilitators. Studies of different applications will allow assessment of the extent to which there are certain determinants that are more important than others. It is also important to investigate the extent to which there are more application-specific barriers and facilitators and whether there are determinants that are more broadly generalizable. Few of the frameworks used in implementation science have yet been applied or “tested” in AI research with regard to their applicability. Determinant frameworks and implementation theories are multi-level, which is important because the implementation of AI in mental healthcare is a socio-technical system that requires conceptualizing the unique, and sometimes asymmetric, contributions of human and technology elements (Holton & Boyd, 2021). Furthermore, applications of AI in metal healthcare can often circumvent traditional workflow and care delivery pathways (Hermes et al., 2019), requiring consideration of how this disruption in traditional care processes necessitates systems-level thinking with regard to implementation.

Implementation science, thus far, has most often focused on implementation of various individual evidence-based practices, typically health interventions with empirical support for their efficacy or effectiveness (Nilsen & Birken, 2020). The implementation and routine use of such practices may require smaller, more incremental changes to professionals’ existing practices than when implementing and deploying a new AI application. AI, on the other hand, is a discontinuous and more disruptive form of change (Scott et al., 2000), likely requiring considerable professional and organizational learning to achieve optimal ways of working.

Implementation strategies to support AI implementation must address barriers specific to implementation in mental health (Graham et al., 2019). Implementation strategies constitute the “how-to” component of changing practice, being methods and techniques that are used to facilitate implementation of evidence-based practices (Curran et al., 2012). Implementation science has documented limited effectiveness for many strategies, which has been attributed to a paucity of the use of theory in the field. However, it could also be that many strategies have been based on inappropriate assumptions about how to achieve practice change. After all, theories are assumptions about a phenomenon, which means that the explanatory power of a given theory depends on the extent to which the assumptions underpinning the theory provide an accurate or plausible explanation of how current practice can be changed (Moore & Evans, 2017). Research is needed to investigate whether the current conceptualization of implementation strategies is sufficient to develop insights and provide the recommendations necessary to inform AI implementation. More careful definition of strategies could be needed, including not only their name, but also the actor and action targets (Leeman et al., 2017). In practice, many implementations involve not one implementation strategy but a bundle of implementation strategies. Given the socio-technical systems involved in AI implementation, strategy bundles are often necessary. Thus, our thinking on implementation strategies needs to adjust to mirror the complexity of AI implementation in mental healthcare.

Implementation outcomes used in implementation science have been defined as the effects of deliberate and purposive actions to implement evidence-based practices (Powell et al., 2012). A range of outcomes has been described: acceptability (is the practice agreeable or satisfactory?); appropriateness (is the practice compatible with the setting, providers or patients?); feasibility (can the practice be used or carried out within the setting?); fidelity (was the practice implemented as intended?); adoption (was there an initial decision or action to try or employ the practice?); sustainability (was the practice maintained or institutionalized within the setting?); penetration (was the practice integrated within the setting?); and cost (what was the cost of implementing the practice?) (Proctor et al., 2010). The concept of outcomes in implementation research is distinct from service system outcomes and clinical patient outcomes. The relevance of various outcomes for AI implementation research needs to be explored (Hermes et al., 2019).

There might also be a need to develop new theoretical approaches or augment and re-contextualize existing ones from implementation science. Greenhalgh et al. (2017) recognized that the present theoretical approaches in implementation science were inadequate to provide satisfactory explanations of the challenges involved in going from small-scale proof-of-concept projects to implementation of new technologies in healthcare. They developed the Non-adoption, Abandonment, Scale-up, Spread, Sustainability framework, which acknowledges the complexity of the environment in which technologies are introduced and accounts for many collective-level influences, including characteristics of the adopter system (e.g., changes in staff roles), the organization's readiness and capacity to innovate, and the wider context.

Despite the obvious potential of using knowledge generated within the field of implementation science to guide AI implementation, a scoping review by Gama et al. (2021) found only seven studies that described various aspects of AI implementation in different healthcare settings. Most of the 2,541 unique articles identified in the searches concerned studies that were not conducted in healthcare settings and/or focused on the earlier steps of the R&D pipeline, e.g., algorithm development, proof-of-concept projects, and AI testing in the laboratory. The authors concluded that “understanding of how to implement AI technology in healthcare practice is still at its early stages of development” (Gama et al., 2021).

In summary, knowledge derived from implementation science could provide an important starting point for research on AI implementation in mental healthcare. This field has generated many insights and provides a broad range of theories, frameworks, and concepts that are likely relevant for the study of AI implementation in mental healthcare. However, when taking advantage of the existing knowledge basis, it is important to also be explorative and study AI implementation in health and mental healthcare as a new phenomenon in its own right since implementing AI may differ in various ways from implementing evidence-based practices in terms of what implementation determinants, strategies, and outcomes are most relevant. Implementation researchers are used to interdisciplinary work and will benefit from collaborating with healthcare professionals, AI developers, and recipients of mental healthcare to generate conceptual, instrumental, and strategic knowledge that can contribute toward realizing the expectations of AI in mental healthcare.
